# The Effect of Soy Protein–Phycocyanin Concentrate Complex Treatment on Biomarkers of HDL Functional Properties in Male Wistar Rats

**DOI:** 10.3390/cimb48010110

**Published:** 2026-01-20

**Authors:** Ilya Vorozhko, Yuliya Sidorova, Nadezhda Biryulina, Sergey Zorin, Nikita Petrov, Tatyana Korotkova, Alla Kochetkova

**Affiliations:** Federal Research Centre of Nutrition Biotechnology and Food Safety, 109240 Moscow, Russia; vorozhko@ion.ru (I.V.); biryulina_nadezhda@mail.ru (N.B.); zorin@ion.ru (S.Z.); petrov-nikita-y@mail.ru (N.P.); tntisha@gmail.com (T.K.); kochetkova@ion.ru (A.K.)

**Keywords:** high-density lipoprotein, HDL fraction, dysfunctional HDL, phycocyanin, soy protein isolate, cholesterol, lipid metabolism

## Abstract

Due to improper nutrition, high-density lipoproteins (HDLs) can be subjected to structural changes, acquiring a dysfunctional phenotype. Therefore, research efforts are currently focused on improving HDL functionality despite its blood level. The aim of this study was to evaluate the effect of phycocyanin concentrate (as part of a food matrix) on the functional properties of HDL. Male Wistar rats were fed a high-fat diet containing 2% cholesterol for 113 days. Experimental animals were treated with 30 and 300 mg/kg b.w. of phycocyanin concentrate mixed with soy protein isolate. Serum and hepatic cholesterol and triglyceride levels, and the content of protein, triglycerides, choline-containing phospholipids, malondialdehyde, sphingosine-1-phosphate, and paraoxonase-1 in HDL fractions were assessed. The decrease in protein in HDL particles is characteristic for dysfunctional phenotype of these particles. Phycocyanin concentrate diet prevented the depletion of protein in HDL particles, regardless of the dosage. The functionality of HDL is associated with paraoxonase-1 activity, which inhibits lipid peroxidation in lipoproteins. Our results have shown a significant increase in the level of paraoxonase-1 in HDL particles in groups treated with phycocyanin. HDL particles become more enriched with triglycerides with the development of hyperlipidemia. Triglycerides in HDL particles and in serum decreased by two times in animals receiving 30 mg/kg b.w. of phycocyanin. The MDA content in HDL particles decreased in all animals receiving a high-fat diet with the addition of 2% cholesterol. The introduction of 300 mg/kg of phycocyanin returned this indicator to the values of the Control group. Thus, biomarkers of dysfunctional changes in HDL in rodent hyperlipidemia models may be a useful tool for assessing lipid metabolism disorders. Also, the results confirm the potential ability to use phycocyanin concentrate as part of lipid-lowering products.

## 1. Introduction

The atheroprotective effect of high-density lipoproteins (HDLs) is primarily based on reverse cholesterol transport (RCT), wherein excess cholesterol from atherosclerotic macrophages/foam cells is transported to the liver for removal and bile excretion [1]. The composition of HDL particles is complex and includes amphipathic lipids (phospholipids and free cholesterol) and surface apolipoproteins, non-polar lipids (cholesterol esters and triglycerides) in the core, and small amounts of other bioactive lipids and biological molecules (e.g., microRNA). HDL particles are considered the most heterogeneous class of plasma lipoproteins due to differences in particle size, charge, and shape [2]. The majority of HDL biological activity is due to their protein content (>50 different proteins), as well as associated bioactive lipids, vitamins, hormones, and microRNAs [3].

HDL may be subjected to structural changes during diseases: long-term hyperglycemia, dyslipidemia, and excessive oxidative stress lead to oxidative and glycation damage of lipids and proteins, resulting in the conversion of HDL into a dysfunctional state. Recent studies have demonstrated that dysfunctional HDL, particularly oxidized HDL, can generate multiple proatherogenic effects in vivo that contradict the antiatherogenic activity of HDL [4].

Therefore, research efforts are currently focused on improving HDL functionality despite its blood level. Changes in the structural components of HDL particles lead to the loss of their biological functions, despite their concentration in the blood [5,6]. Accordingly, methods for preserving HDL functionality and preventing their transformation into a proinflammatory phenotype are of significant scientific and practical interest. One of such possible methods may be the use of proteins with hypocholesterolemic activity, like phycocyanins.

Phycocyanin (PC) is a protein widely used as blue food coloring, consisting of two non-covalently linked subunits, α and β. The main source of phycocyanin is blue–green algae, belonging to the genus *Arthrospira*. The antioxidant, hypoglycemic, and hypocholesterolemic properties of *Arthrospira platensis* (spirulina) biomass extracts have been widely studied in vitro, in vivo, and in clinical trials [7].

For example, a clinical study [8] involving 40 patients with metabolic syndrome assessed the effects of a commercial spirulina extract containing phycocyanin. Consumption of the extract at a dose of 20 mg PC/day significantly decreased serum triglyceride levels and increased HDL levels in these patients. Spirulina extract consumed for 2 weeks at a dose of 11 mg C-phycocyanin (C-PC)/kg b.w. per day with drinking water by male golden Syrian hamsters treated with a high-fat diet already decreased the level of low-density lipoproteins (LDLs) in the animals’ blood, as well as the hepatic concentration of non-esterified fatty acids and cholesterol [9]. Intragastric administration of C-PC to male Swiss mice at a high dose of 500 mg/kg b.w. for 16 weeks, treated with a high-fat diet, led to a significant decrease in the concentration of proinflammatory cytokines (TNF-α and IL-6) and leptin in brown adipose tissue [10]. Consumption of *Spirulina maxima* extract at doses of 150 and 450 mg/kg b.w./day by male ICR mice treated with a high-fat diet for 6 weeks led to a significant decrease in body weight gain, blood triglyceride, cholesterol, and LDL levels. The extract, only at a dose of 450 mg/kg b.w./day, contributed to an increase in serum HDL level in the experimental animals [11].

It is known that some antioxidants in isolated form may exert either proantioxidant effects due to excessive consumption or have limited effectiveness due to poor intestinal absorption [12,13]. For example, the interaction of polyphenolic antioxidants with macromolecules, especially proteins, has been studied for the purpose of concentration and immobilization on the food matrix [14,15,16]. SPI is characterized by a high amino acid profile (PDCAAS coefficient > 75), contains all essential amino acids, and has its own set of potential biological effects, which are also widely covered in the scientific literature. About 70% of SPI proteins are globulins, in particular 7S (β-conglycinin), consisting of three subunits weighing 50–70 kDa, and 11S (glycinin), which includes five polypeptide subunits weighing 20–35 kDa [17]. In combination with soy protein isolate, polyphenols were found to have improved hypoglycemic activity, bioavailability, and digestibility [14]. Soy protein has been extensively studied for its preventive role in various diseases. Soy protein has high biological value and contains biologically active isoflavones, which can reduce hepatic lipid levels. Studies [18,19,20] have shown that soy protein consumption can reduce serum cholesterol and triglyceride levels, and reduce the accumulation of hepatic cholesterol and triglycerides, thereby reducing liver steatosis. In a series of studies [21], obese Zucker Rats treated with soy protein enriched with isoflavones (6.7% of the diet, for 6 weeks) had reduced hepatic fat content and blood ALT and AST levels, upregulated hepatic beta-oxidation processes, and decreased SREBP-1 mRNA levels, leading to reduced hepatic triglyceride and cholesterol levels in obese diabetic Zucker rats.

Thus, the aim of this study was to evaluate the effect of low and high doses of phycocyanin concentrate (as part of a food matrix) on the functional properties of high-density lipoproteins in male Wistar rats consuming a high-fat diet containing 2% cholesterol.

## 2. Materials and Methods

### 2.1. Obtaining the Phycocyanin Concentrate

We previously developed a method for obtaining such concentrates with a total PC content of 32% and a purity level of 1.0 [22].

Briefly, a 60 g sample of spirulina biomass was extracted with 600 mL of 0.1 M potassium phosphate buffer, pH 7.0, pre-cooled to +4 °C for 8 h at a temperature of +4 °C, with constant stirring on a magnetic stirrer. The resulting suspension was then centrifuged (Beckman J-6B centrifuge, Brea, CA, USA) at 4560× *g* for 30 min. The supernatant was separated from the sediment by decantation, the sediment was re-extracted using a similar scheme, and the extracts were combined. The combined extract was subjected to tangential flow ultrafiltration through a membrane with a pore diameter of 30 kDa, with collection of the high molecular weight fraction and subsequent freeze-drying.

### 2.2. Determination of Phycocyanin Content

The content of C-phycocyanin (C-PC) and allophycocyanin (A-PC) in the concentrate was quantitatively assessed by determining the optical density at wavelengths of 620 nm and 655 nm and using Formulas (1) and (2) for calculation [22]:

(1) $$C_{c-pc}=\frac{\left(0.154\times OD620-0.1\times OD655\right)}{m\times (1-k)\times l}\times V\times 100\%$$

(2) $$C_{a-pc}=\frac{\left(0.194\times OD655-0.034\times OD620\right)}{m\times (1-k)\times l}\times V\times 100\%$$

Notes: *C_c-pc_*—concentration of C-phycocyanin, %; *C_a-pc_*—concentration of allophycocyanin,%; *V*—volume of combined extract, mL; *m*—sample weight, mg; *k*—water mass fraction in the sample; *l*—optical path length, cm.

The purity degree of the obtained concentrate was calculated based on the optical density ratio OD620/OD280.

### 2.3. Animals

The experiment was conducted over 113 days on 60 growing male Wistar rats (age ~3 weeks) with an initial body weight of 50 ± 5 g.

Animal studies were carried out in accordance with standard principles described in the “Guide for the Care and Use of Laboratory Animals” [23] and approved by the local ethics committee (Protocol No. 11 dated 15 December 2021). Animals were housed 2 rats per cage under controlled environmental conditions (temperature was 20–26 °C, relative humidity was 30–60%, and with a 12 h lighting cycle).

### 2.4. Experimental Design

The behavioral phenotype of animals was studied before the experiment to divide them into active and passive species. The preliminary division of animals according to their behavior increases the repeatability of obtained results [24,25]. After 7 days of acclimatization, 5 days before the start of the experiment, the behavioral indicators of each rat were determined in the open field (OF) test. In the open field test, an animal is placed in an unfamiliar open space from which it cannot escape. An unfamiliar environment triggers a complex set of behavioral reactions that reflect anxiety and the desire to explore new territory, i.e., the animal’s behavior in the OF is determined by the ratio of defensive and exploratory tendencies. Testing was carried out under standard lighting conditions for 3 min (180 s). During testing, the following behavioral indicators were recorded: the number of zone transitions, time spent in each zone, and distance traveled. The movement of animals across the field was recorded using the Smart 3.0.04 software (Panlab, Barcelona, Spain).

**Figure 1:**
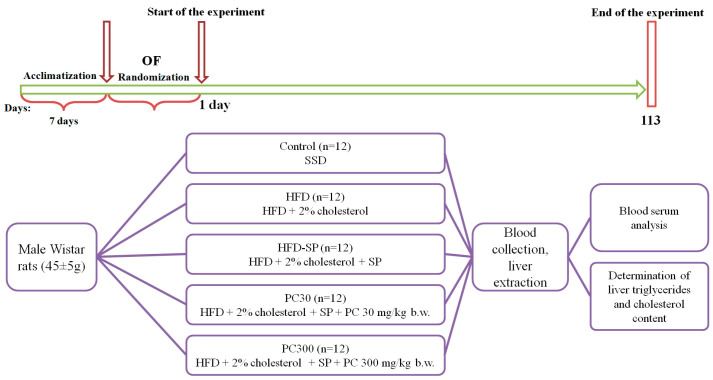
Experimental design. Note: OF—open field, SSD—semi-synthetic diet, HFD—high-fat diet, SP—soy protein isolate, and PC—phycocyanin concentrate.

Three days before the start of the experiment, the animals were weighed, and their blood glucose levels were determined. Based on body weight, blood glucose level, and OF test results, the animals were divided into 5 groups: Control (*n* = 12), HFD (*n* = 12), HFD-SP (*n* = 12), PC30 (*n* = 12), and PC300 (*n* = 12). The experimental design is shown in Figure 1.

Animals of the Control group received a standard semi-synthetic diet (SSD) throughout the experiment. Animals of the HFD group received a high-fat diet with the addition of 2% cholesterol: (HFD + 2% cholesterol diet). Animals of the group HFD-SP received a high-fat diet with 2% cholesterol, in which 50% of the protein component was replaced by Supro 760 IP soy protein isolate (Solae Belgium, Zwaanhofweg, Belgium). Animals of groups PC30 and PC300 received the same high-fat diet as animals of group HFD-SP, to which phycocyanin concentrate was added at a dose of 30 and 300 mg/kg of body weight, respectively (Table 1). Phycocyanin concentrate was preliminarily mixed with soy protein isolate, forming a food matrix, which was added to the HFD diet.

All animals received food and drinking water ad libitum during the experiment. On day 113, animals were decapitated under light anesthesia. Blood collected after decapitation was incubated at 2–8 °C for 3 h, centrifuged for 30 min at 2560× *g* at 4 °C, and the resulting serum was stored at −20 °C. The liver was removed by pathological dissection and weighed.

Parameters of lipid metabolism (total cholesterol, HDL, and triglycerides) were determined in blood serum using the automatic biochemical analyzer “Konelab 20i” (ThermoScientific, Waltham, MA, USA).

To study biomarkers of dysfunctional changes in HDL, particles from blood serum were obtained by sequential ultracentrifugation in KBr saline solutions according to the method [27] with some modifications aimed at adapting the technique for using an Optima MAX-XP ultracentrifuge with a TLA 110 rotor (Beckman Coulter, Brea, CA, USA). A 4.5 mL whole blood sample was centrifuged for 5 min at 2000× *g* +20 °C. Plasma was collected and centrifuged for 5 min at 13,000× *g* +20 °C, and then 1 mL of infranatant was collected without touching the chylomicron layer, in a test tube with 325 mg of dry KBr and mixed until completely dissolved. In total, 3.6 mL of KBr solution with d = 1.210 was layered on the sample and centrifuged for 17 h at 266,000× *g* at +8 °C. 0.7 mL of the floating fraction was collected and mixed with 1.4 mL of a 0.9% chloride solution at +20 °C. 2.5 mL of KBr solution with d = 1.063 was layered, and the mixture was centrifuged again for 17 h at 266,000× *g* at +8 °C. A part of the sample was collected from the meniscus, leaving 1 mL of infranatant, and mixed until completely homogenized.

The particles were assayed for protein, cholesterol, triglyceride, choline-containing phospholipids (CCLPs), and malondialdehyde (MDA) using biochemical methods, as well as paraoxonase-1 (PON1) and sphingosine-1-phosphate (S1P), using ELISA. The content of analytes relative to HDL cholesterol was calculated. The quality of fraction extraction was assessed by the correlation between the HDL value, determined by the enzymatic colorimetric method with immunoinhibition in blood serum, and the fraction cholesterol.

The content of malondialdehyde in blood serum was determined using the competitive ELISA method according to the manufacturer’s method (Elabscience, Houston, TX, USA).

Fat was extracted from the liver using the Folch method according to [28]. The content of triglycerides and cholesterol in fat extracted from the liver was determined photometrically using the Konelab 20i automatic biochemical analyzer (ThermoScientific, USA).

### 2.5. Statistical Analysis

For intergroup comparisons, the Kolmogorov–Smirnov goodness-of-fit test with a normal distribution was calculated. If the asymptotic significance of the criterion was less than or equal to 0.05, the distribution pattern was considered to be different from normal, and intergroup comparisons were performed using the Kruskal–Wallis test (data presented as Me (Q1–Q3)). If the asymptotic significance of the criterion was greater than 0.05, the distribution pattern was considered normal, and intergroup comparisons were performed using ANOVA (data presented as mean ± SEM). Intergroup differences were considered statistically significant at *p* ≤ 0.05. In case of detection of statistically significant differences between groups, a post hoc pair-wise Dunnett test was performed, in which group 1 was considered the Control. For the Dunnett test, differences from the Control group were considered statistically significant at *p* ≤ 0.05.

## 3. Results

### 3.1. Characterization of Phycocyanin Concentrate

The table shows the results of determining the content of C-PC and A-PC in the concentrate, and the total yield (%) of these phycobiliproteins from the *A. platensis* biomass used (Table 2).

### 3.2. Results of In Vivo Study

The general condition of the animals of the Control group and the experimental groups HFD-SP, PC30, and PC300 in terms of appearance and fur quality during daily examination was satisfactory. The animals in group HFD had greasy and tousled fur.

Serum HDL levels in groups PC30 and PC300 were significantly decreased compared to the Control group (Table 3).

Consumption of a high-fat diet supplemented with 2% cholesterol resulted in a significant decrease in the relative protein content in HDL particles. The relative protein content in HDL particles was significantly higher in HFD-SP and PC30 groups, compared to the HFD group, and the difference with the Control group was not significant. There were no significant differences in the animals of the PC300 group compared with both the Control and the HFD group.

In all animals receiving a high-fat diet, the relative content of choline-containing phospholipids (CCPLs) in HDL particles significantly reduced compared to the Control group. At the same time, this indicator was significantly higher in animals of the PC300 group compared to the HFD-SP and PC30 groups.

The level of paraoxonase-1 in HDL particles significantly increased in animals of the HFD-SP and PC30 groups; the differences were significant compared to animals of the Control and HFD groups. The tendency to increase in PON1 level was observed in animals of the PC300 group.

The significantly higher level of serum MDA was found in animals of the HFD, HFD-SP, and PC300 groups compared to the Control group (Table 3 and Table 4). Despite this, all animals receiving a high-fat diet had significantly decreased relative content of MDA in HDL particles compared to the Control group. The addition of phycocyanin concentrate at a dose of 30 mg/kg b.w. prevented the excessive accumulation of MDA only in the blood serum; no significant differences with the Control group were found.

**Figure 2:**
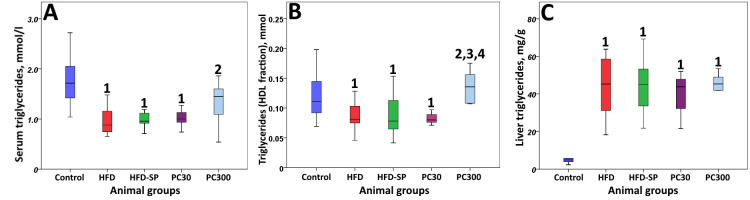
Triglyceride content in serum (**A**), in HDL particles (**B**), and in liver (**C**). Note: 1—differences are significant compared to Control group (*p* < 0.05); 2—differences are significant compared to HFD group; 3—differences are significant compared to HFD-SP group (*p* = 0.01); 4—differences are significant compared to PC30 group (*p* = 0.01); and HDLs—high-density lipoproteins.

The accumulation of triglycerides in the liver of animals treated with a high-fat diet supplemented with 2% cholesterol was accompanied by a decrease in the triglyceride content of HDL particles in the HFD, HFD-SP, and PC30 groups (Figure 2). We obtained similar results for the content of triglycerides in HDL particles relative to cholesterol (Table 4).

On the contrary, the addition of phycocyanins at a dose of 300 mg/kg b.w. led to significant (two-fold) accumulation of triglycerides in HDL particles. We observed the dose-dependent effect against the background of a tendency to an increase in serum triglycerides.

**Figure 3:**
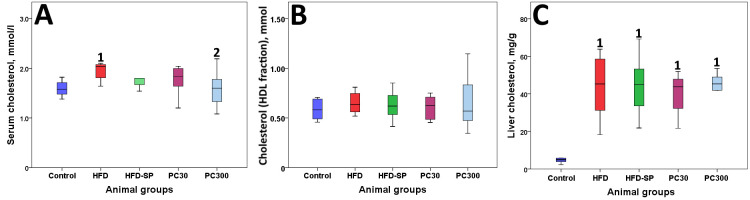
Cholesterol content in serum (**A**), in HDL particles (**B**), and in liver (**C**). Note: 1—differences are significant compared to Control group; 2—differences are significant compared to HFD group; (*p* < 0.05), and HDLs—high-density lipoproteins.

Consumption of a high-fat diet with the addition of 2% cholesterol led to a significant increase in blood and hepatic cholesterol levels (Figure 3). There were no significant differences in cholesterol content in HDL fractions. The introduction of soy protein isolate and a complex of soy protein isolate with phycocyanin concentrate prevented the excess accumulation of cholesterol in blood, while consuming high doses of phycocyanins made the difference significant. However, no effect on hepatic cholesterol levels was detected.

## 4. Discussion

In our study, we investigated the effect of soy protein isolate itself and as a food matrix mixed with phycocyanin concentrate in low (30 mg/kg b.w.) and high (300 mg/kg b.w.) doses in the composition of a high-fat diet with the addition of 2% cholesterol on male Wistar rats. The interaction of biologically active compounds (BACs) with proteins depends on both the structure of the BAC and the structure of the protein, as well as the pH of the solution, ionic strength, temperature, and the BAC/protein ratio [29]. During binding, hydrophobic interactions of aromatic groups of amino acid residues of the protein and the BAC and/or hydroxyl groups of the BAC with the protein chain may occur [30]. Such binding may cause a change in the spatial structure of the protein and affect the activity of both components.

Consumption of a high-fat diet supplemented with 2% cholesterol by animals of the HFD group resulted in decreased relative protein content in HDL particles. That is characteristic for dysfunctional phenotype of these particles and depletion of adaptive potential. The introduction of soy protein isolate in the diet, both individually and in combination with phycocyanin, prevented the depletion of protein in HDL particles. A similar result was obtained in [31]. Male ApoE^−/−^ mice consumed polyphenol-containing extract of black elderberry (1.25% in the diet) for 6 weeks, which led to an improvement in the functional state of HDL, as expressed in a significant increase in the PON1 activity and content.

In a clinical study [32], healthy women consumed acai berries, 200 g/day, for 4 weeks. Increased blood levels of ApoA1 and PON1 activity were observed, which, according to the authors, characterizes improved functional properties of HDL.

Along with the accumulation of lipid peroxidation (LPO) products in the blood, a decrease in the MDA content in HDL particles was noted in all groups of animals receiving a high-fat diet with the addition of 2% cholesterol, except for animals of the PC30 group.

Accordingly, consumption of phycocyanin concentrate in a dose of 30 mg/kg b.w. by animals prevented the accumulation of MDA in the blood serum, exerting a certain antioxidant effect. The introduction of soy protein isolate alone and as a food matrix mixed with phycocyanin had no effect on MDA content in the HDL fraction.

It is likely that the content of LPO products in HDL particles depends more on the lipidome of particles that perform the RCT than on the intensity of LPO processes in the animal’s body or the liver acceptor function.

The relative content of CCPLs in HDL particles was reduced in all groups compared to the Control. That characterizes the changes in the lipidome of HDL particles due to disruption of RCT. It seems that these changes are due to the consumption of a high-fat diet with added 2% cholesterol by animals. The study [33] also found a decrease in the content of phospholipids in the HDL composition in the blood of patients with non-alcoholic steatohepatitis. The authors suggest that one of the possible reasons for this effect may be decreased phospholipid biosynthesis in the liver, which is consistent with our data showing an accumulation of cholesterol and triglycerides in the animals’ liver against the background of an increase in liver fat. No significant effect of soy or phycocyanin on this indicator was observed in the experiment. However, with the addition of PC 300 mg/kg b.w., a tendency towards an increase in the level of CCPLs in HDL particles was revealed: the differences were significant compared to the HFD-SP and PC30 groups (*p* < 0.05). It was found that in the animals of the PC30 group, which received phycocyanin at a dose of 30 mg/kg b.w., the CCPL content in the particles directly correlated with the MDA content (r^2^ = 0.75, *p* < 0.001), which was not observed either in the Control group (r^2^ = −0.07) or in the HFD group (r^2^ = 0.13). At the same time, when phycocyanin was administered at a high dose (300 mg/kg b.w., PC300 group), this relationship was lost. Since CCPLs are major components of the HDL surface monolayer and are involved in the process of removing cellular cholesterol into particles, the obtained data may indicate that the administration of phycocyanin at a dose of 30 mg/kg b.w. led to CCPLs-mediated acceptance of MDA from cells.

The antioxidant, antiatherogenic, and cardioprotective effects of HDL are associated with PON1 activity, which is responsible for inhibiting lipid peroxidation by hydrolyzing phospholipid hydroperoxides and cholesterol esters in lipoproteins. PON1 binds to HDL through interaction with ApoA1 and phospholipids. It can protect HDL from oxidative modification and thus may inhibit the progression of atherosclerosis [34]. Our result has shown a significant increase in the level of paraoxonase-1 in HDL particles in HFD-SP and PC30 groups, and a tendency for its increase in PC300 group. Similarly, in the work [35], a significant increase in the expression of PON1 was revealed in male Wistar rats that were treated intragastrically with nano-formulated spirulina methanol extract at a dose of 100 mg/kg b.w. for 21 days compared to the Control group.

Other studies also showed beneficial effects of natural antioxidants on improving HDL functionality. For example, in the study [36], intragastric administration of curcumin at a dose of 340 mg/kg b.w. to Wistar rats with HgCl2-induced oxidative stress led to a significant increase in PON1 activity and a decrease in LDL oxidation susceptibility. Consumption of acai berries rich in polyphenolic compounds by female Fischer 344 rats at a dose of 2 g/day for 6 weeks against the background of a high-fat diet contributed to an increase in serum and hepatic PON1 activity, and also stimulated hepatic expression of ApoA1 [37].

The enrichment of HDL with triglycerides occurs as a result of exchange mediated by the cholesterol ester transfer protein with TG-rich lipoproteins [38]. Thus, the transfer of TG from the pool of triglyceride-rich lipoproteins to HDL is associated with the outflow of cholesterol esters from HDL to TG-rich lipoproteins [39], making HDL a more suitable substrate for lipolysis by hepatic lipase, an enzyme that plays a key role in HDL metabolism [40]. As a result, the accumulation of TG in HDL particles can enhance the lipolytic transformation and subsequent metabolic clearance of these particles [41]. With the development of hyperlipidemia in humans, HDL particles become more enriched with triglycerides, which replace the cholesterol ester in the lipoprotein core, thus acquiring pro-inflammatory, pro-oxidative, and pro-thrombotic properties.

In our study, on the contrary, a significant decrease in this indicator was observed in the HFD group receiving a high-fat diet with cholesterol, and in the HFD-SP and PC30 groups compared to the Control group. Only in animals fed a high dose of phycocyanins with soy protein isolate was this difference not significant. Moreover, hepatic triglycerides in all experimental groups were significantly increased, while blood triglycerides, on the contrary, were decreased. This is probably how the heterogeneity of the structure, composition, and biological functions of HDL in rats and humans is manifested.

### Limitations of the Study

The first limitation of this study is that we used the approach of a ten-fold step between doses of food matrix (30 and 300 mg/g in terms of phycocyanin) and the absence of average doses. The second limitation is the absence of an animal group with phycocyanin concentrate added without soy protein.

## 5. Conclusions

The study presented in this paper is of a pilot nature. The obtained results show a pronounced influence of a high-fat diet on markers of the functional state of HDL, demonstrating their transition to a dysfunctional form. Moreover, phycocyanins in the food matrix exerted a protective effect on HDL particles by regulating the levels of protein and paraoxonase in HDL. Interesting data on triglyceride metabolism along the HDL-liver axis have been obtained, which may become the subject of further, more in-depth research.

Thus, biomarkers of dysfunctional changes in HDL in rodent hyperlipidemia models may be a useful tool for assessing lipid metabolism disorders. Our results confirm the prospects for using phycocyanin concentrate as part of a functional food matrix of protein nature.

## Figures and Tables

**Table 1:** Composition of experimental diets.

Components	Content, g/100 g of Diet				
	Control	HFD	HFD-SP	PC30	PC300
Casein	23.8	23.8	11.9	11.9	11.5
Soy protein isolate	-	-	10.4	10.4	10.4
Sunflower oil	5.0	5.0	5.0	5.0	5.0
Porcine lard	5.0	23.7	23.7	23.7	23.7
Corn starch	60.8	20.1	21.6	21.6	19.0
Sucrose	-	20.0	20.0	20.0	19.0
Mixture of minerals * (g)	4.0	4.0	4.0	4.0	4.0
Vitamins lipid-soluble * (mL)	0.1	0.1	0.1	0.1	0.1
Vitamins water-soluble * (g)	1.0	1.0	1.0	1.0	1.0
L-cysteine	0.3	0.3	0.3	0.3	0.3
Cholesterol	-	2.0	2.0	2.0	2.0
Phycocyanin concentrate	-	-	-	0.45	4.8
Caloric value, kcal	410 ± 5	499 ± 5	505 ± 5	499 ± 5	490 ± 5

Note: *—According to AIN-93 [26].

**Table 2:** The content of C-PC and A-PC in the concentrate.

Sample	C-PC % by Weight	A-PC % by Weight	Σ_(C-PC+A-PC)_	Purity Degree, OD620/OD280	Yield, % Σ_(C-PC+A-PC)_
PC	31.8 ± 0.6	5.2 ± 0.2	37.0 ± 0.8	1.04	64.9 ± 1.1

Notes: PC—phycocyanin concentrate; C-PC—C-phycocyanin; A-PC—allophycocyanin; and OD620/OD280—optical density at wavelength 620 or 280 nm, respectively.

**Table 3:** Serum levels of HDL and MDA.

Parameter	Animal Groups				
	Control	HFD	HFD-SP	PC30	PC300
HDL, mmol/L	1.05 ± 0.04	0.96 ± 0.06	0.92 ± 0.03	0.88 ± 0.04 ^1^	0.88 ± 0.06 ^1^
MDA, ng/mL	935 (524–1055)	1838 (1605–2580) ^1^	1782 (963–3028) ^1^	1195 (993–1452)	3253 (1062–5019) ^1^

Notes: HDLs—high-density lipoproteins, MDA—malondialdehyde, and ^1^—differences are significant compared to Control group. *p* < 0.05.

**Table 4:** Biomarkers of dysfunctional changes in HDL (relative content per 1 mmol of HDL–cholesterol).

Parameter	Animal Groups				
	Control	HFD	HFD-SP	PC30	PC300
Protein, g	1.03 ± 0.12	0.67 ± 0.06 ^1^	0.92 ± 0.08 ^2^	0.93 ± 0.05 ^2^	0.90 ± 0.07
Triglycerides, mmol	0.19 (0.17–0.30)	0.14 (0.11–0.16) ^1^	0.14 (0.11–0.17) ^1^	0.13 (0.12–0.17) ^1^	0.26 (0.21–0.33) ^2,3,4^
CCPLs, mol	0.90 (0.85–0.93)	0.71 (0.69–0.75) ^1^	0.69 (0.65–0.72) ^1^	0.65 (0.60–0.70) ^1^	0.74 (0.72–0.82) ^1,3,4^
S1P, mg	400 (341–457)	328 (290–382)	338 (265–410)	325 (279–415)	428 (260–472)
PON1, ng	42 (39–116)	82 (36–221)	216 (126–579) ^1,2^	306 (157–434) ^1,2^	159 (101–202)
MDA, μmol	124.6 (120.2–131.6)	92.6 (88.5–105.6) ^1^	108.7 (98.1–113.3) ^1^	92.6 (86.4–109.4) ^1^	99.0 (96.8–120.5) ^1^

Notes: CCPLs—choline-containing phospholipids, S1P—sphingosine-1-phosphate, PON1—paraoxonase-1, MDA—malondialdehyde, ^1^—differences are significant compared to Control group, ^2^—differences are significant compared to HFD group, ^3^—differences are significant compared to HFD-SP group, and ^4^—differences are compared to PC30 group. *p* < 0.05.

## Data Availability

The original contributions presented in this study are included in the article. Further inquiries can be directed to the corresponding author.

## Abbreviations

The following abbreviations are used in this manuscript:

ALT	alanine transaminase
A-PC	allophycocyanin
ApoA1	apolyprotein A1
AST	aspartate transaminase
BAC	biologically active compound
CCPLs	choline-containing phospholipids
C-PC	C-phycocyanin
HDL	high-density lipoprotein
HFD	high-fat diet
IL-6	interleukin-6
LDL	low-density lipoprotein
LPO	lipid peroxidation
MDA	malondialdehyde
PC	phycocyanin
PON1	paraoxonase 1
RCT	reverse cholesterol transport
S1P	sphingosine 1 phosphate
SP	soy protein isolate
TGs	triglycerides
TNF-α	tumor necrosis factor α
